# Is there malaria transmission in urban settings in Colombia?

**DOI:** 10.1186/s12936-015-0956-0

**Published:** 2015-11-14

**Authors:** Julio C. Padilla, Pablo E. Chaparro, Karen Molina, Myriam Arevalo-Herrera, Sócrates Herrera

**Affiliations:** Ministerio de Salud y Protección Social de Colombia, Bogotá, Colombia; Instituto Nacional de Salud, Bogotá, Colombia; Malaria Vaccine and Drug Development Center, Cali, Colombia; Faculty of Health, Universidad del Valle, Cali, Colombia; Centro de Investigación Científica Caucaseco, Cali, Colombia

**Keywords:** Epidemiology, Malaria, Peri-urban, Urban population, Insect vectors

## Abstract

**Background:**

Colombia contributes a significant proportion of malaria cases in the Americas, which are predominantly rural. However, in the last 8 years ~ 10 % of the endemic municipalities have also reported urban and peri-urban malaria cases, a growing concern for health authorities. This study focused on the characterization of the officially reported urban malaria cases.

**Methods:**

A descriptive retrospective study based on secondary information provided by the Colombian National Surveillance System-SIVIGILA for the 2008–2012 period was conducted. A total of 17 municipalities with consistent and persistent reports of urban and peri-urban malaria were selected for analysis, which included site of origin and of residence, age, gender and ethnicity of patients, health system affiliation, *Plasmodium* species and the presence of malaria vectors.

**Results:**

A total of 18,113 malaria cases were reported from urban and peri-urban areas of 17 endemic municipalities. Almost 70 % of the reports originated in localities in the departments of Chocó and Nariño, located on the Pacific Coast where a predominantly Afro-Colombian population, of individuals of under 30 years of age, was the most affected (80.7 %), mainly with *Plasmodium falciparum* infections (52.1 %). Median annual parasite index (API) was 6.4 per 1000 inhabitants (3.4 in 2008; 10.8 in 2010 and 6.0 in 2012). Between 2011 and 2012 complicated cases (2.4 %) and malaria in pregnant women (1.4 %) were reported. Study areas reported the presence of at least seven *Anopheles* species considered malaria vectors. These analyses did not allow ascertaining the presumable origin of the recorded urban cases due to the lack of a consensus on a definition of urban, peri-urban and rural limits and the lack of proper verification of the geographical source of infection.

**Conclusions:**

The study indicates the probable presence of endemic, unstable and low-intensity malaria transmission in Colombian urban and peri-urban areas of a group of municipalities located mainly on the Pacific coast region and a few others in the eastern region. There is a need to unequivocally confirm the urban or peri-urban origin of the malaria cases reported and the transmission conditions, as well as to develop and implement new strategies for urban and peri-urban malaria control and elimination.

## Background

Malaria remains an important global health problem that affects mainly poor communities in Africa, Asia and Latin America, with an estimated ~198 million clinical malaria cases and ~584,000 deaths reported worldwide in 2013 [1]. Approximately 80 % of these cases were caused by *Plasmodium falciparum*, ~20 % by *Plasmodium vivax,* and a more limited number of cases by the other parasite species, including *Plasmodium malariae* and *Plasmodium ovale* [1]. Although the great majority of malaria cases occur in rural areas worldwide, during the last 30 years a growing number of urban and peri-urban cases has been reported, and a number of systematic reviews have shown the impact of urbanization on malaria transmission, mainly in sub-Saharan African countries [2–4], where the annual entomological inoculation rates (EIR) from dozens of African cities have shown an increasing gradient from urban to peri-urban and to rural areas [2]. Likewise in Asia, malaria reemergence in countries, such as India has been associated with rapid peri-urban expansion and an increase in poverty in cities such as Mumbai, New Delhi and Chennai [5, 6]; meanwhile malaria vectors have been found to have adapted to the urban context in this country [7–9].

In Latin America, Brazil has shown a population movement tendency to urban and peri-urban areas of cities in the Amazon region due forest colonization, road construction and the establishment of agricultural and mining settlements during the last decades of the 20th century, with an enormous impact on malaria transmission [10, 11]. Between 1986 and 2005 the population in Manaus increased in the form of slums and housing projects displaying an increase in disease incidence and the annual parasite index (API) from low to medium risk and, in urban zones, from no risk to high risk [12, 13]. In Rôndonia, another Brazilian Amazon region, the population increased 12 times between 1970 and 1990, with a consequent 30-fold increase in malaria cases in that period [14]. Anarchical urban colonization and the presence of malaria vectors and asymptomatic carriers led to peri-urban malaria transmission in cities from this region [14, 15].

A similar situation exists in other Latin American countries, such as Peru, Ecuador and Colombia [9, 16, 17]. In Peru, several regions have reported peri-urban malaria transmission: Iquitos (an Amazonian city) [18], Sullana and Piura (in the Northwestern Pacific Coast) [19] and Lima (the capital city) [20]. Also Esmeraldas (San Lorenzo) in Northern Ecuador has confirmed peri-urban malaria transmission [21]. In Colombia, this problem is poorly understood, but previous studies have suggested the presence of urban and peri-urban malaria transmission foci of both *P. vivax* and *P. falciparum* in towns such as Quibdó, Buenaventura and Puerto Gaitán [22–24]. In all these countries peri-urban areas are characterized by rapid and anarchical urbanization induced mostly by population migration from rural endemic settings to areas with the presence of different *Anopheline* species. This rapid urbanization of population with poor socio-economic conditions, inadequate housing infrastructure and lack of public services, usually leads to improper sanitation, poor drainage of surface water and the consequent development of water bodies and vegetation-rich areas [22, 23]. These are suitable ecological conditions for mosquito breeding and malaria transmission. Additionally, some mosquito species appear to easily adapt to new breeding conditions in urban settings, such as water containers.

Moreover, in Colombia multiple factors, such as armed conflict, the presence of paramilitary groups, and illegal agriculture and mining, among others, significantly contribute to the displacement of rural populations to peri-urban areas in several regions. One such region is the Colombian Pacific coast, which has been reporting increasing urban and peri-urban malaria transmission [25, 26]. This region with a length of ~1300 km is located between Panama and Ecuador, and includes the departments of Chocó, Valle del Cauca, Cauca and Nariño and is characterized by tropical rain forests, with the highest national rainfall amounts (3000–12,000 mm annually) and is also rich in natural resources but displays the poorest social and economic development of the country. Ecological conditions in this region also favor the presence of several species of *Anopheles* mosquitoes, including important malaria vectors, such as *Anopheles albimanus, Anopheles nuneztovari s.l.* and *Anopheles darlingi*, which appear to have the ability to colonize a variety of breeding sites, adapting to changing environments including urban and peri-urban areas [18, 27–29]. Other regions where urban and peri-urban malaria has been reported are Puerto Gaitán and Villavicencio, both located in the Eastern Region, where ecological conditions for malaria are present and the armed conflict influences the migration of population from rural endemic settings to urban/peri-urban dwellings. Few studies have addressed the problem at local scale in Colombia [22–24, 30] with the consequent gaps in knowledge on the real extent of urban and peri-urban malaria at national scale.

Despite growing and improving statistics and surveillance, it looks like a proportion of reported cases are actually not real urban or peri-urban cases, but the result of an erroneous classification and registration of cases, greatly due to problems regarding the following: (1) “urban setting” and “urban malaria” definitions, (2) origin of malaria cases, (3) lack of identification of mosquito breeding sites, and (4) lack of confirmation of the vector´s transmission capacity in urban settings. These factors cause uncertainty about the true extent and importance of urban or peri-urban malaria transmission. Solving these issues would be critical to adjusting the strategies for malaria prevention and control in urban areas.

In conclusion, it appears that in contrast to the overall malaria decreasing trend at national level, the number of cases in urban settings is increasing and the growth rate is greater in urban than in rural settings. In response to the Millennium Development goals, Colombia has included the goal towards reducing malaria urban transmission in two recent government strategic policies: The Ten-Year Plan for Public Health (2012–2021) and the Integrated Management Strategy for Promotion, Prevention and Control of Vector-Borne Diseases (EGI-ETV) [31]. To contribute to this goal, this study has attempted to identify and characterize the epidemiology of urban and peri-urban malaria transmission in Colombia between 2008 and 2012, to establish a baseline for the current initiative of the Colombian government to significantly reduce malaria by 2021 [32].

## Methods

### Type of study

A descriptive study was carried out using information obtained from records compiled by SIVIGILA, from January 1, 2008 to December 31, 2012. Information was collected using standard notification forms in which the malaria clinic is assigned to the municipality defined as the geographical area bounded by the urban perimeter as defined by an administrative agreement [33] and includes both urban and peri-urban areas, as opposed to well-defined rural areas and with no correlation with population density.

Case definition was established according to the SIVIGILA protocol in which a malaria case is a patient having visited malaria endemic areas, in this case urban or peri-urban settings, in the 15 days prior to the current or recent febrile episode (>37.5 °C) with *Plasmodium* infection confirmed either by thick blood smear (TBS) or rapid diagnostic test (RDT).

### Study sites

**Fig. 1:**
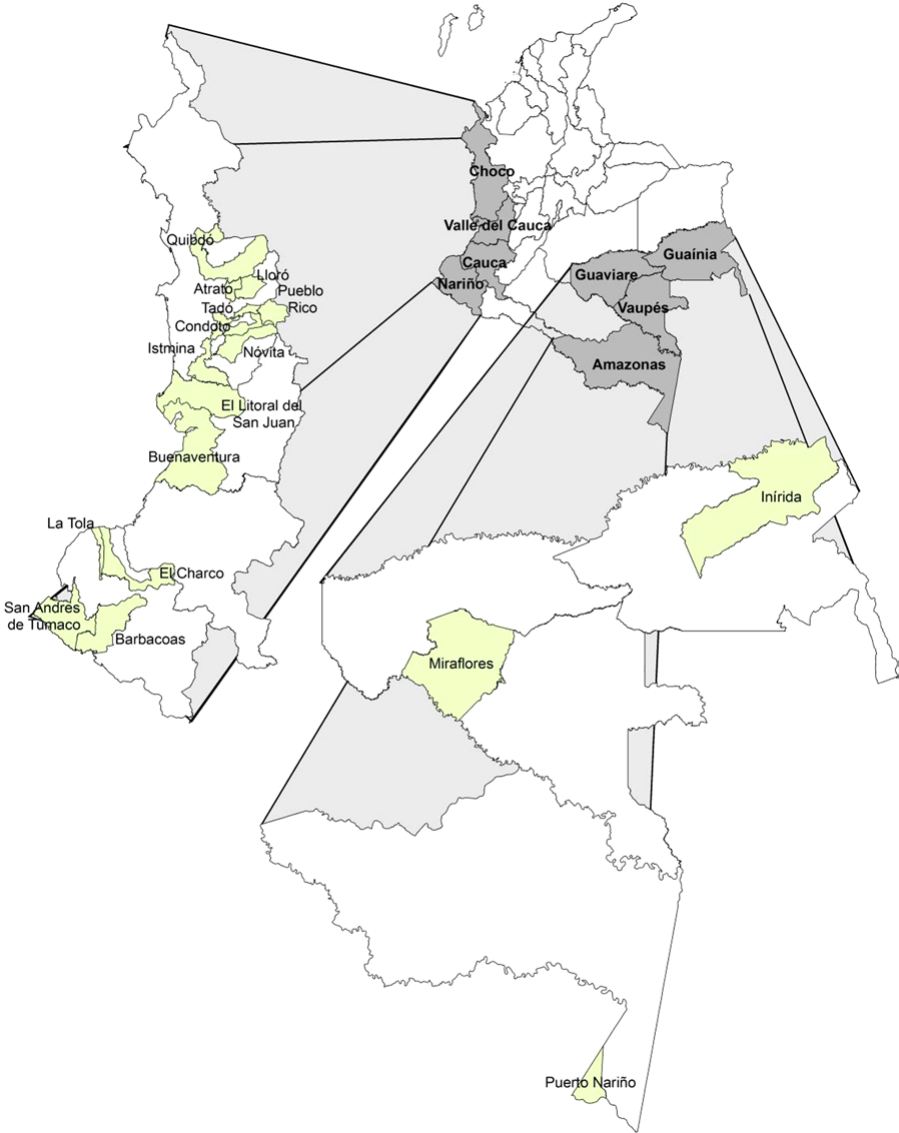
Main municipalities reporting urban and recent peri-urban malaria transmission in Colombia since 2008–2012

Localities included in the analysis were administrative units (municipalities) corresponding to urban and peri-urban areas with consistent and persistent malaria cases reports to SIVIGILA. Most of these localities have been historically reported as sites with urban transmission foci and reported entomological evidence of the presence of malaria mosquito vectors according to information from the vector borne diseases (VBD) Prevention and Control Programme of the Colombian Ministry of Health (MOH). The study focused on a total of 13 municipalities of departments located along the Pacific coast as follows: eight municipalities located in department of Chocó (Atrato, Condoto, Istmina, Litoral del Bajo San Juan, Lloró, Nóvita, Quibdó and Tadó); four in Department of Nariño (Barbacoas, El Charco, La Tola and Tumaco); and one in department of Valle del Cauca (Buenaventura). Four municipalities scattered in several departments of the central and Eastern regions of the country were also included in the analysis, namely: Pueblo Rico in department of Risaralda (central region), Puerto Inírida, in department of Guainía, Miraflores in department of Guaviare (Eastern region), and Puerto Nariño, in Amazonas department (Southern region) (Fig. 1).

### Study variables

Variables included were age, gender, ethnicity and type of health service affiliation, *Plasmodium* species, department and municipality of origin and of residence during the year the malaria episode was reported. Severe malaria cases and malaria in pregnant women were also included.

### Vector information

Information regarding the presence of malaria vectors in each selected municipality with urban malaria was obtained from the field-work report of the Colombian National Institute of Health (Instituto Nacional de Salud, INS) and from previous publications [22, 23, 27, 28, 34].

### Statistical analyses

Epidemiological and entomological variables were processed using Microsoft Excel^®^. Univariate analysis were performed using the software Tableau statistical software version 6.0^®^. A univariate analysis was performed for all variables. Measures of central tendency and dispersion were calculated. The API was calculated and urban population values used for each municipality were obtained from the projections of the 2005 Colombian census [35]. In order to compare the malaria morbidity profile, yearly 2008–2012 APIs were compared using a two-sample *T* test at a 0.05 significance level to assess statistically significant differences between *P. falciparum* and *P. vivax* cases.

## Results

**Fig. 2:**
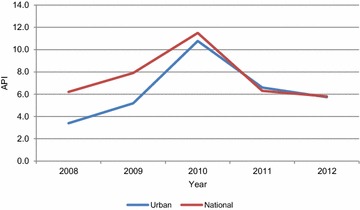
Malaria incidence in Colombia from 2008 to 2012. Incidence of malaria is shown as annual parasite index (malaria cases per 1000 inhabitants) for urban settings (*blue line*) and the whole country (national, *red line*)

In total, 18,113 malaria cases classified as urban or peri-urban by SIVIGILA were recorded from 2008 to 2012 with an annual average of 3623 cases, a maximum of 6133 in 2010 and a minimum of 1853 in 2008. These cases corresponded to extreme APIs of 10.8 in 2010 and 3.4 in 2008, and an average API of 6.4 cases per 1000 inhabitants for the study period. The peak for 2010 correlates with the national malaria outbreak reported that year [36] (Fig. 2). Additionally, these cases accounted for 3.0 % of the total malaria cases in Colombia in 2008 and 5.9 % of cases in 2011. A moderate decline in API was observed in the 2010–2012 period. The majority of the cases (52.2 % or 9431), were caused by *P. falciparum,* whereas 46.1 % (8356) were *P. vivax* infections, with an additional 1.6 % (293) of cases due to mixed *falciparum*/*vivax* infections and 0.02 % (three cases) considered non-indigenous *P. malariae* cases.

Over 66 % of the urban and peri-urban malaria cases were reported in children and adults <30 years of age, with the highest incidence reported in the 20–29 age group. From this age on, cases started decreasing and the lowest incidence was reported for individuals aged over 60. Men represented 57.3 % of the malaria cases reported. The comparison between *Plasmodium* species showed that the API was higher in men (6.4 cases per 1000) than in women (4.8 cases per 1000) with *P. vivax* predominant (61.2 %) among men and *P. falciparum* among women (44.3 %) (Table 1).

**Table 1:** Main features of urban and peri-urban malaria cases reported in Colombia by *Plasmodium* species from 2008 to 2012

Variable	*P. falciparum* (*n* = 9431)		*P. vivax* (*n* = 8356)		*P*
	*n*	%	*n*	%	
Age group					
0–9	1450	15.3	1513	18.1	0.04
10–19	2644	27.9	1993	23.9	<0.01
20–29	2204	23.3	2080	24.9	0.22
30–39	1301	13.8	1167	14.0	0.89
40–49	954	10.1	859	10.3	0.89
50–59	524	5.5	461	5.5	1.00
60–69	236	2.5	192	2.3	0.89
70–79	110	1.2	78	0.9	0.84
80 years+	38	0.4	13	0.2	0.92
Sex					
Female	4193	44.3	3245	38.8	<0.001
Male	5268	55.7	5111	61.2	<0.001
Ethnic group					
Indigenous	229	2.4	609	7.3	<0.01
Afro, mulato	8632	91.2	5985	71.6	<0.001
Other	599	6.3	1761	21.1	<0.001
Insurance					
Affiliated	6926	73.2	6300	75.4	<0.01
Not affiliated	2532	26.8	2054	24.6	0.09

**Fig. 3:**
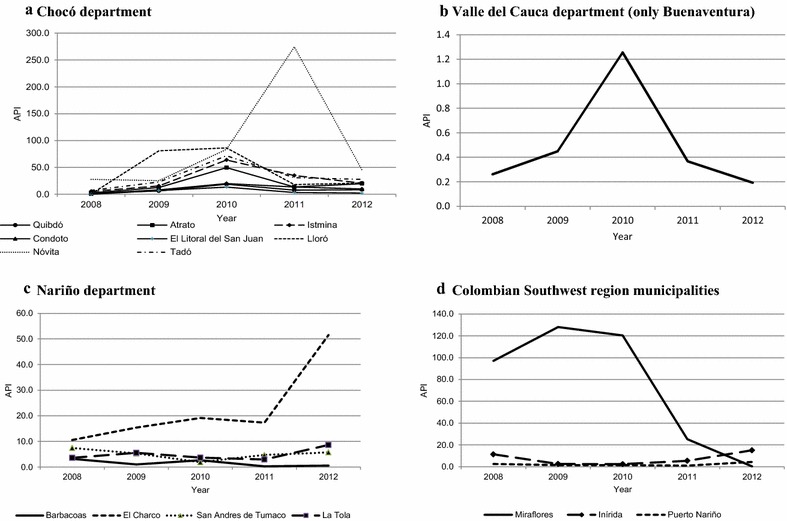
Annual parasite index in Colombian municipalities reporting urban and peri-urban transmission from 2008 to 2012. **a** Department of Chocó, **b** Department of Valle del Cauca (only Buenaventura), **c** Department of Nariño and **d** Colombian Southwest region municipalities

The municipalities with the highest cumulative burden of malaria cases were Quibdó (Chocó) (4536 cases), Istmina (Chocó) (2702 cases), Tumaco (Nariño) (2441 cases), Tadó (Chocó) (1898 cases) and Nóvita (Chocó) (1357 cases), which contributed about 70 % of the total urban and peri-urban cases for the study period. However, the transmission behaviour of urban malaria in each one of these areas showed a variable trend. Novita (Chocó) presented the highest API in 2011 whereas Barbacoas (Nariño) and Buenaventura (Valle del Cauca) displayed the lowest API during this period. Tumaco (Nariño) presented with a hypo-endemic and decreasing transmission tendency. Miraflores (Guaviare) recorded a dramatic and sustained decline in the urban and peri-urban transmission trend (Fig. 3).

Istmina, Tadó and El Charco showed the highest annual *P. falciparum* malaria indexes (>14 cases per 1000 inhabitants), whereas Miraflores and Novita exhibited the highest annual *P. vivax* malaria indexes (>47 cases per 1000 inhabitants) (Table 2). Afro-descendant population was the most affected, accounting for 80.7 % of the cases, whereas indigenous communities accounted for 4.6 % of the cases. The remaining cases (14.7 %) occurred in ethnic minority groups.

**Table 2:** Annual parasite indexes of the municipalities reporting urban malaria in Colombia from 2008 to 2012

	***AFI***	***AVI***	*API*
Chocó			
Nóvita	44.5	47.9	93.0
Lloró	5.9	33.8	41.4
Tadó	14.1	17.8	32.2
Istmina	15.5	12.2	28.3
Atrato	11.3	8.3	19.9
Condoto	3.6	6.8	10.6
Quibdó	5.1	3.2	8.6
El Litoral del San Juan	2.2	3.0	5.2
Nariño			
El Charco	21.6	1.9	23.5
San Andrés de Tumaco	3.9	1.1	5.0
La Tola	4.0	1.0	5.0
Barbacoas	1.0	0.6	1.5
Guaviare			
Miraflores	10.1	59.7	71.0
Guainía			
Puerto Inírida	0.2	7.1	7.4
Amazonas			
Puerto Nariño	0.0	2.2	2.2
Risaralda			
Pueblo Rico	0.1	2.0	2.1
Valle del Cauca			
Buenaventura	0.1	0.4	0.5

*AFI* annual falciparum index, *AVI* annual vivax index, *API* annual parasitic index

A total of 174 of the 7242 cases (2.4 %) reported in 2011 and 2012, were classified as complicated malaria cases, most of which (146 or 83.9 %) originated in Chocó, whereas the departments of Valle del Cauca and Nariño reported 19 and 7 cases, respectively. Moreover, during this period a total of 100 cases occurred in pregnant women of whom 69 % were from Choco, 18 % from Nariño, and 10 % from Valle del Cauca.

The analysis of entomological data available from the Colombian INS indicates that predominant *Anopheles species* were: *An. albimanus*, *An. darlingi*, *Anopheles neivai*, *Anopheles nuneztovari,Anopheles lepidotus, Anopheles pseudopunctipennis*, and *Anopheles punctimacula,* which in general were widely distributed in peri-urban areas and most of them overlapped in the different study settings [23, 27, 28], as well as a few *Anopheles spp* in the urban area of Quibdó [22] (Table 3).

**Table 3:** Entomological information

Region	Department	Municipality	*Anopheline* species	References
Pacific coast	Chocó		*An. nuneztovari* *An. neivai* *An. darlingi* *An. albimanus* *An. pseudopunctipennis* *An. punctimacula* *An. lepidotus* *Anopheles spp.*	[21, 22]
	Valle del Cauca	Buenaventura	*An. nuneztovari* *An. albimanus* *An. neivai* *An. pseudopunctipennis* *An. albimanus*	[21, 23, 27, 28]
	Nariño		*An. neivai* *An. albimanus* *An. pseudopunctipennis* *An. punctimacula*	[21]
Orinoquia and Amazonia	Guainía Guaviare	Puerto Inírida Miraflores	*An. darlingi*	[21, 34]
	Amazonas		*An. darlingi* *An. nuneztovari*	[21]
	Risaralda	Pueblo Rico	*An. albimanus* *An. pseudopunctipennis*	[21]

## Discussion

This study aimed to characterize the official reports on existence of peri-urban malaria transmission in different areas in Colombia, with greater relevance in important municipalities of the Pacific coast known for the highest malaria transmission indexes in the country. Peri-urban malaria was also reported with lower indexes in areas of the central and Eastern regions of the country. In general, malaria transmission presented with a low-intensity and variable pattern, limited to areas near urban centers in a small group of municipalities.

Although at national level the overall malaria transmission presented a higher proportion of *P. vivax* cases (~70 %), the distribution of the reported urban and peri-urban cases displayed an equivalent proportion of *P. falciparum* and *P. vivax* cases. This is explained by the fact that most reported cases were from Chocó and Nariño departments, where the population is mainly composed of African descendant communities with a high prevalence of Duffy negative individuals [37]. The lack of the Duffy Antigen Receptor for Cytokines (DARC) on the red blood cell membrane prevents the development of *P. vivax* blood infections while allowing the normal invasion and development of *P. falciparum* [38–40].

Our results, based on the official SIVIGILA records and Colombian INS data, are in agreement with previous studies conducted in Colombia in which other approaches and data sets were used [22, 23, 26, 28, 41] as well as with studies in other endemic countries in the Americas [9, 10, 13, 15]. Studies carried out in the urban area of Buenaventura (Valle del Cauca) between 1987 and 1993 found that urban malaria had a tendency towards quadrennial occurrence of epidemic peaks with targeted distribution of cases and a greater proportion of cases in men. However, the prevalence of *P. falciparum* infection was significantly greater and the most affected groups were those of extreme ages (<5 and >60 years of age) [23], whereas in our study malaria cases were more prevalent in the 20–29 years of age range and the lowest incidence was after 60 years of age.

Previous studies have found that the main breeding sites for malaria peri-urban vector were located in excavations left by the miners, as well as lakes and ponds dedicated to fish farming where immature *An. nuneztovari* and *An. albimanus* mosquito species with peri-domiciliary adult bite rates up to 7.1 were also found [28]. Even though a study in urban areas of Quibdó (Chocó) found 839 positive cases recorded as urban malaria cases (77 % of which were due to *P. falciparum*), only 24.4 % of these were confirmed as indigenous urban malaria cases [22]. Malaria transmission appeared to be focused on peri-urban areas for both *P. falciparum* and *P. vivax*, with the greatest risk of transmission in proximity to areas of vegetation and excavations left by miners. The occurrence of malaria in children, students and housewives suggests that malaria transmission takes place inside households and at school neighborhoods [22]. Other focal studies such as the one conducted in Puerto Gaitán in the department of Meta located in the Eastern region of Colombia have reported similar results. In Puerto Gaitán 70 % of the 192 malaria cases reported in 2009 were from urban areas; whereas in 2010 malaria cases increased to 226 with only 46 % being urban [24, 42].

There is a contrast between the decreasing trend of rural malaria and the stable or increasing records on urban malaria. This might be explained by multiple conditions that could favor the introduction of malaria into peri-urban and urban areas. In Colombia population movements from endemic rural areas is mostly due to the ongoing armed conflict, but more recently also to the dissemination of illegal mining and proliferation of illicit crops; all of these generate fear and threaten safety in rural communities. Additionally, the lack of incentives for rural communities to remain in their regions as well as the apparent stimulus by the government for urban population with the hope for better jobs, encourage the migration from rural to urban and peri-urban areas. The migration of malaria infected individuals contributes to generate conditions for the introduction and maintenance of *Plasmodium* transmission [43] in peri-urban and urban areas. A recent study showed a significant number of malaria asymptomatic carriers who were able to efficiently infect mosquitoes despite the presence of low sub-microscopic parasitaemia [37].

Interestingly, in this study records of complicated malaria cases as well as malaria in pregnant women were found. In a previous study carried out in some of the same localities, most febrile patients in endemic areas attended the diagnostics service within 5 days of the onset of malaria symptoms [21, 44] which may help prevent the development of complicated cases [45] Additionally, during the last two decades, Colombia has significantly increased its social security coverage, which would be expected to result in improved prenatal follow-up [46].

This study aimed at a better characterization of the reported urban and peri-urban malaria transmission and could serve as a reference transmission baseline at national scale to be further used for monitoring activities to evaluate compliance with the goal established by the INS-ETV (Vector-borne diseases) of eliminating urban and peri-urban malaria in Colombia by 2021. Although urban areas display different exposure risks and offer better opportunities for prevention and timely control due to the existing infrastructure, technological capabilities and access to health services [3, 47, 48], defining urban and peri-urban areas remains a challenge. The study was based on the SIVIGILA records in which the urban and peri-urban concepts are defined based on administrative factors that not necessarily take into consideration the ecology i.e., the presence of water collection, mosquitoes breeding sites, bushes and other more rural conditions. This limitation, as well as the need to unify operational criteria for improving the quality and consistency of information, improving the malaria detection system, and timely control and monitoring supported by a geographic information system, should be addressed to unequivocally confirm the extent of urban and peri-urban malaria in Colombia. Besides the lack of an appropriate definition of urban, peri-urban and rural limits in most endemic areas studied, reliability and quality of available information are affected by a potential reporting bias due to case misclassification induced by the lack of proper verification of the geographical source of infection. Patient information is not always reliable [21, 25, 48] or there may be bad processing of the mandatory format for reporting infectious diseases. For example, for safety reasons patients avoid providing information about the potential site of infection frequently related to areas of illegal activities. In addition, malaria control officers may not receive appropriate instruction or be aware of the critical importance of properly recording the geographical origin of the infection. Moreover, frequently the place of occurrence or origin of the case is confused with the place of temporal or permanent residence or the case report site.

Although malaria in urban residents has been conclusively shown, it remains critical to determine the extent of local transmission, including the definition of associated entomological factors such as presence of mosquito breeding places, abundance and behaviour of adult *Anopheles.* Environmental factors including precipitation, relative humidity and temperature also affect mosquito breeding [28] and must be further studied.

## Conclusions

Despite the limitations described, this study documents the probable presence of endemic, unstable and low-intensity urban and peri-urban malaria cases in a group of municipalities located mainly on the Pacific coast region and a few others in the Eastern region. This study explored some key epidemiological factors related to malaria transmission in the urban settings selected to set up the grounds for ongoing prospective studies aimed at unequivocally confirming the urban or peri-urban origin of the malaria cases and the transmission conditions to aid in achieving the goal of urban malaria elimination in Colombia by 2021.
